# Supplementary material for: Comparative genomics provides new insights into the diversity, physiology, and sexuality of the only industrially exploited tremellomycete: Phaffia rhodozyma
Source: BMC Genomics. 2016 Nov 9;17:901. doi: 10.1186/s12864-016-3244-7 (PMC5103461; doi:10.1186/s12864-016-3244-7)
Supplement: Additional file 6: — List of orphan genes with links to PFAM (related to Additional file 1: Table S1). (ZIP 1428 kb) [file 12864_2016_3244_MOESM6_ESM.zip › BLAST_HTML_FTR/G04670_P.html]

BLAST Search Results


```
BLASTP 2.2.27+


Reference:
Stephen F. Altschul, Thomas L. Madden, Alejandro A. Schäffer,
Jinghui Zhang, Zheng Zhang, Webb Miller, and David J. Lipman (1997),
"Gapped BLAST and PSI-BLAST: a new generation of protein database
search programs", Nucleic Acids Res. 25:3389-3402.


Reference for
composition-based statistics:
Alejandro A. Schäffer, L. Aravind, Thomas L. Madden, Sergei
Shavirin, John L. Spouge, Yuri I. Wolf, Eugene V. Koonin, and
Stephen F. Altschul (2001), "Improving the accuracy of PSI-BLAST
protein database searches with composition-based statistics and
other refinements", Nucleic Acids Res. 29:2994-3005.


Database: nr
           71,551,133 sequences; 26,053,659,533 total letters


Query= G04670_P

Length=144
                                                                      Score     E
Sequences producing significant alignments:                          (Bits)  Value

emb|CED83744.1|  hypothetical protein [Xanthophyllomyces dendrorh...   291    2e-98
ref|XP_004386398.2|  PREDICTED: oral-facial-digital syndrome 1 pr...  40.4    0.27 
ref|WP_049570207.1|  hypothetical protein [Nonomuraea sp. SBT364]     36.6    5.4  


 >emb|CED83744.1| hypothetical protein [Xanthophyllomyces dendrorhous]
Length=143

 Score =  291 bits (746),  Expect = 2e-98, Method: Compositional matrix adjust.
 Identities = 143/143 (100%), Positives = 143/143 (100%), Gaps = 0/143 (0%)

Query  1    MYKISTSPPVFSKYNVGDRVLFYPHPSQPEESHHQPCSSSTSPRTIPSTKPQQVSTPCTR  60
            MYKISTSPPVFSKYNVGDRVLFYPHPSQPEESHHQPCSSSTSPRTIPSTKPQQVSTPCTR
Sbjct  1    MYKISTSPPVFSKYNVGDRVLFYPHPSQPEESHHQPCSSSTSPRTIPSTKPQQVSTPCTR  60

Query  61   EKRRDSSNSDQLTGPDNSEVSTGVVEKVQVIPAQYVEGGPFSSSPPGWRGIVETDGINYL  120
            EKRRDSSNSDQLTGPDNSEVSTGVVEKVQVIPAQYVEGGPFSSSPPGWRGIVETDGINYL
Sbjct  61   EKRRDSSNSDQLTGPDNSEVSTGVVEKVQVIPAQYVEGGPFSSSPPGWRGIVETDGINYL  120

Query  121  IKEDRSGLAAVYNDDEIVSLTGT  143
            IKEDRSGLAAVYNDDEIVSLTGT
Sbjct  121  IKEDRSGLAAVYNDDEIVSLTGT  143


>ref|XP_004386398.2| PREDICTED: oral-facial-digital syndrome 1 protein [Trichechus 
manatus latirostris]
Length=1050

 Score = 40.4 bits (93),  Expect = 0.27, Method: Composition-based stats.
 Identities = 20/56 (36%), Positives = 29/56 (52%), Gaps = 0/56 (0%)

Query  27   SQPEESHHQPCSSSTSPRTIPSTKPQQVSTPCTREKRRDSSNSDQLTGPDNSEVST  82
            SQP  +  +P  SS SPR++P  K          + R + SN D+LT  DN E+ +
Sbjct  811  SQPSPAESRPTPSSASPRSLPEQKASLYQKQAELQDRSEFSNLDKLTSKDNEELDS  866


>ref|WP_049570207.1| hypothetical protein [Nonomuraea sp. SBT364]
Length=578

 Score = 36.6 bits (83),  Expect = 5.4, Method: Composition-based stats.
 Identities = 24/57 (42%), Positives = 32/57 (56%), Gaps = 9/57 (16%)

Query  63   RRDSSNSDQLTGPDNSEVSTGVVEKV--QVIPAQYVEGGPFSSSPPGWRGIVETDGI  117
            RRDS+ SD LTGP N++V  GVV ++  +  P   V GGP       W G+  T G+
Sbjct  217  RRDSAGSDALTGP-NADVVPGVVRRLLGETFPTGAV-GGPLR-----WGGVTATGGL  266


Lambda      K        H        a         alpha
   0.313    0.131    0.392    0.792     4.96 

Gapped
Lambda      K        H        a         alpha    sigma
   0.267   0.0410    0.140     1.90     42.6     43.6 

Effective search space used: 638910511260


  Database: nr
    Posted date:  Sep 23, 2015 12:05 AM
  Number of letters in database: 26,053,659,533
  Number of sequences in database:  71,551,133


Matrix: BLOSUM62
Gap Penalties: Existence: 11, Extension: 1
Neighboring words threshold: 11
Window for multiple hits: 40
```
